# A copper foam-based surface-enhanced Raman scattering substrate for glucose detection

**DOI:** 10.1186/s11671-023-03776-x

**Published:** 2023-02-09

**Authors:** Wang Peng, Zhihan Xu, Xiangting Jia, Qingxi Liao

**Affiliations:** 1 College of Engineering, Huazhong Agricultural University, Wuhan, 430070 China; 2 Shenzhen Branch, Guangdong Laboratory for Lingnan Modern Agriculture, Genome Analysis Laboratory of the Ministry of Agriculture, Agricultural Genomics Institute at Shenzhen, Chinese Academy of Agricultural Sciences, Shenzhen, 518000 China; 3 Shenzhen Institute of Nutrition and Health, Huazhong Agricultural University, Wuhan, 430070 China; 4 Key Laboratory of Agricultural Equipment in Mid-Lower Yangtze River, Ministry of Agriculture and Rural Affairs, Wuhan, 430070 China

**Keywords:** Copper foam, Surface-enhanced Raman scattering, Glucose detection

## Abstract

Raman spectroscopy can quickly achieve non-destructive, qualitative and quantitative detection, and analysis the molecular structure of substances. Herein, a facile and low-cost method for preparation of highly sensitivity SERS substrates was implemented through the displacement reaction of copper foam immersed in AgNO_3_ ethanol solution. Due to the 3D structure of copper film and homogenous displacement, the Ag–Cu substrate showed high performance SERS enhancement (1.25 × 10^7^), and the lowest detection concentration for R6G reached 10^–10^ Mol/L. For glucose detection, mixed decanethiol (DT)/mercaptohexanol (MH) interlayer was used to enable glucose attach to the substrate surface, and the limit of detection reached to 1 uM/L. SERS substrate makes the Ag–Cu SERS substrate promising for biological applications.

## Introduction

Surface-enhanced Raman scattering (SERS) can achieve ultra-sensitive detection of target molecules, and it has been used in various professional fields [1–4]. Due to its label-free analysis capability, SERS can be applied in various fields such as food safety, environmental monitoring, biomedicine, agricultural, military, and explosives testing [5–10].

Currently, researchers attribute surface-enhanced Raman scattering to two mechanisms: electromagnetic enhancement and chemical enhancement. In the electromagnetic enhancement, the localized electromagnetic field enhancement caused by surface plasmon resonance is considered to be the most important contribution. Surface plasmon is the collective oscillation effect of free electrons in the metal under the photoelectric field, which greatly increases the strength of the electromagnetic field. The chemical enhancement is attributed to the fact that when molecules are chemically adsorbed on the surface of the substrate, surface adatoms and other co-adsorbed species may have certain chemical interactions with the molecules, and the change of the conversion rate affects its Raman intensity [11–13].

Researchers have developed many nanomaterials suitable for SERS, such as classic noble metal nanomaterials (gold and silver) [14–16]. Silver nanoparticle have attracted widespread attention due to its advantages of surface plasmon resonance, dielectric properties and chemical stability. Thus, silicon wafers and polymer films have been used as substrate for nanoparticles to enlarge its SERS functional area, yet the deposition or evaporation of nanoparticles on these substrates requires expensive equipment and complex fabrication process [17–23]. Therefore, it is necessary to develop a low-cost and good performance SERS substrate with a simple fabrication process.

Nowadays, many advanced SERS functional layer forming processes have been developed. Hu et al. [24] reacted and electrochemically deposited a layer of metallic silver on copper foil, and studied the growth process of silver dendrites during the reaction; Li et al. [25] prepared ITO glass by dropping silver nanowires on ITO glass- Ag substrate; Ma et al. [26] adsorbed a layer of gold nanoparticles on PDMS membrane to prepare PDMS-Au membrane; Mhlanga et al. [26] obtained high enhancement factors by in situ growth of gold and silver layers on silicon wafers and glass slides.

A facile and low-cost method for preparation of highly sensitivity SERS substrates was implemented through the displacement reaction of copper foam immersed in AgNO_3_ ethanol solution. With the substitution reaction between Cu and silver nitrate, a layer of silver nanostructures is deposited on the surface of Cu in a certain period of time.

Over the years, various analytical methods based on mass spectrometry, electrochemistry, chemiluminescence, fluorescence, and colorimetry have been used to achieve sensitive and selective detection of glucose. Optical methods have been extensively studied due to their advantages of minimally invasive or non-invasive, portability, relatively low-cost, sensitivity and selectivity [27–31]. Several optical techniques have been developed to detect glucose, such as infrared absorption, laser polarization, dye fluorescence modification, bioimpedance spectroscopy, and thermal emission spectroscopy. Most of these techniques are not molecularly specific and can yield similar results with structurally similar molecules [32–38]. In order to enhance the detection ability and specificity of biomolecules, the SERS substrate had been functionalized to make it better to adsorb glucose molecules.

In this work, a facile and low-cost method had been demonstrated for the preparation of high-sensitivity SERS substrates through the displacement reaction of copper foam immersed in AgNO_3_ ethanol solution. By adjusting the reaction time, silver dendrites with different morphologies were observed on the substrate surface. In a certain reaction time, the Ag–Cu substrate showed the best SERS enhancement by comparing the enhancement effect, and the lowest detection concentration of Rhodamine 6G was as low as 10^−10^ M. For glucose detection, mixed decanethiol (DT)/mercaptohexanol (MH) interlayer brings glucose closer to the substrate surface with detection limits as low as 1 uM.

## Methods and measurements

### Materials and reagents

Copper foam (thickness 0.5 mm) was provided by Guangzhou Lige Company. Silver nitrate (AgNO_3_, ≥ 99%), acetone (≥ 99%), ethanol (≥ 99%), dilute sulfuric acid(H_2_SO_4_, ≥ 99%), Rhodamine 6G (R6G),1-DT, and MH were purchased from the laboratory. The water used in all the experiments was purified in the Millipore Milli-Q manner. All materials were of high purity and were used as received without further purification or treatment.

### Fabrication of Ag–Cu SERS substrate

**Fig. 1:**
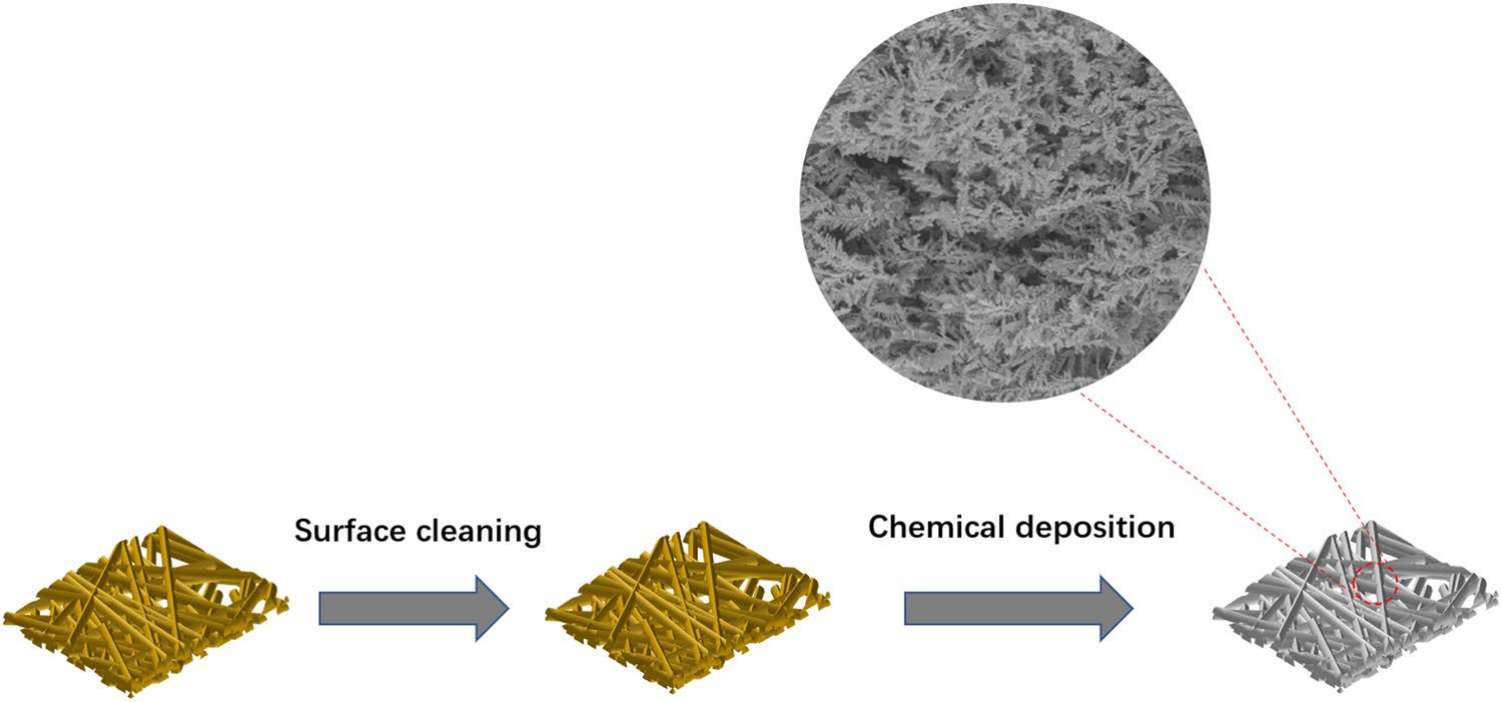
SERS substrate fabrication flow chart

Firstly, a reaction solution is prepared. At room temperature, silver nitrate powder were dissolved in ethanol solution, and AgNO_3_ ethanol solution with a concentration of 10 mM was prepared to react with copper foam. The copper foam was cut into small pieces of 1 cm × 1 cm and washed with acetone, ethanol and deionized water in turn to remove organic impurities. To obtain a fresh copper foam surface, the cleaned copper foam was soaked in 1% dilute sulfuric acid to remove surface oxides, and then thoroughly rinsed with deionized water. The pretreated copper foam was soaked in AgNO_3_ and ethanol mixed solution, due to the standard electro-chemical potential of Ag (0.80 V) higher than that of Cu (0.34 V), Ag^+^ could be reduced to elemental Ag by obtaining electrons from Cu of the foamy copper. Ag nuclei firstly formed on the surface of foamy copper surface and then grew into Ag nanoparticles which construct Ag dendrite, then silver nanostructures were formed on the copper foam through galvanic displacement reaction. The reaction time was controlled at 4 min, 6 min, and 8 min, respectively. The final Ag–Cu substrate was rinsed with deionized water, then pure dried with nitrogen, placed on a drying table to evaporate the water, and then used for SERS testing. The fabrication process of the SERS substrate is shown in Fig. 1.

### Instruments and measurements

Analytical investigations of size and morphology were performed by a scanning electron microscope (HITACHI, SU8010 FE-SEM). All SERS spectra in this paper were measured and recorded on a confocal Raman microscope system (DXR3 Raman Microscope), equipped with an excitation wavelength of 785 nm. Besides, drying operation was performed in a Drying table (DB-XAB, China).

### SERS detection of analytes

Firstly, a series of analytes standard solutions were prepared according to different concentration gradients. R6G solutions of different concentrations were dropped on the prepared substrate. As the solution was dried, the Rhodamine molecules were adsorbed on the substrate for detection. For glucose detection experiments, SERS substrates were incubated in ethanol with 1 mM DT for 45 min and then transferred to ethanol with 1 mM MH for at least 12 h. Then the SERS substrate functionalized surface was dripped with glucose solution for SERS measurement. The spectra were measured with the 785 nm laser and acquired after two cumulative scans, each scan time was 10 s. More than three points were randomly selected on each sample for testing, and the average value was calculated.

## Results and discussion

### Characterization of SERS substrate

**Fig. 2:**
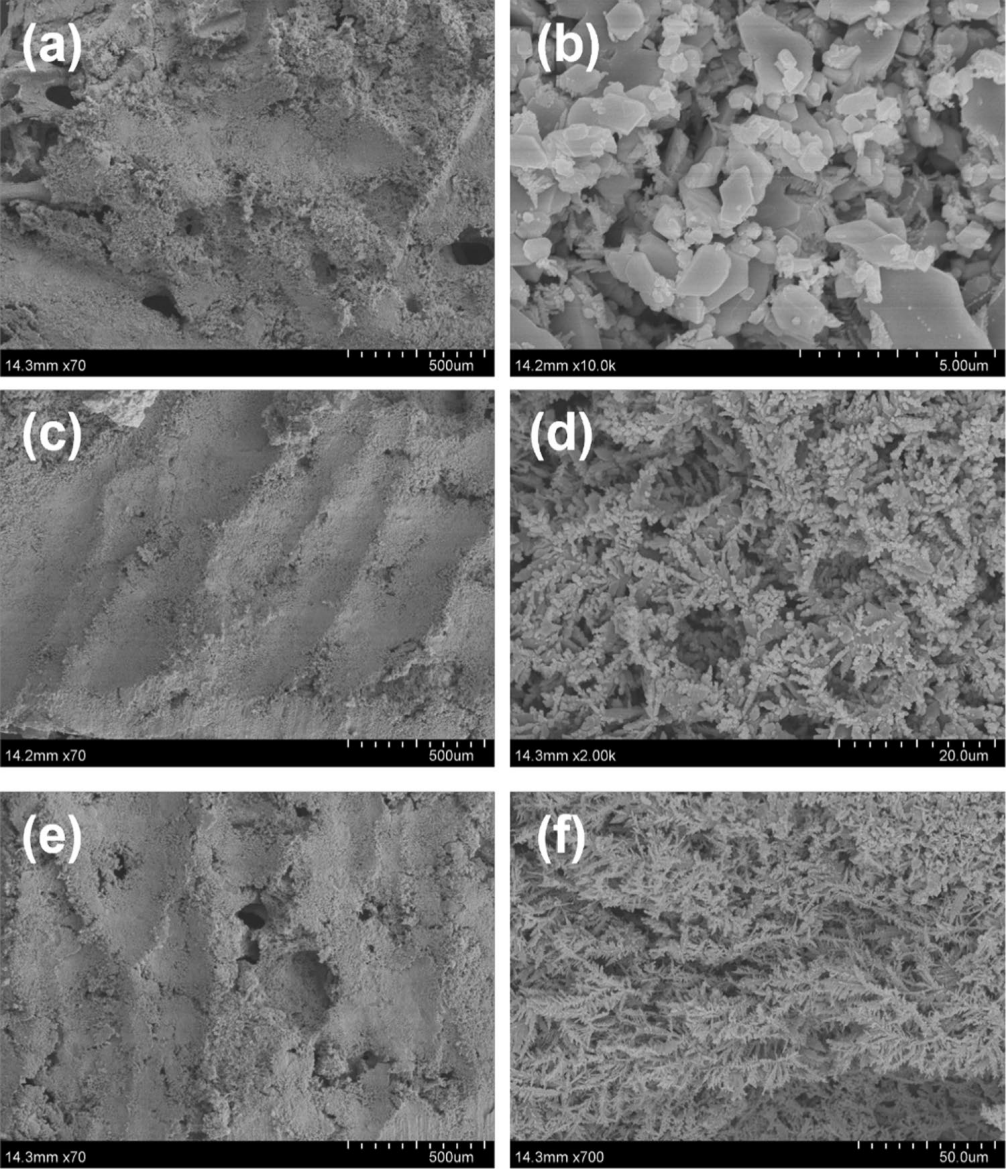
Scanning electron microscope images of silver dendrites on the surface of copper foam by electrochemical deposition of silver nitrate for 4 min (**a**, **b**), 6 min (**c**, **d**), and 8 min (**e**, **f**)

A series of microstructural characterization analysis after the sample was synthesized, and its scanning electron microscope image is shown in Fig. 2. It shows the growth of silver dendrites on the surface of copper foam observed by field emission scanning electron microscopy. After 4 min reaction, the silver particles covered the gaps of the copper foam, and the shape was sheet-like (Fig. 2a, b). With the increase of the reaction time, the initial formation of silver dendrites can be clearly observed at the reaction time of 6 min (Fig. 2c, d). When the reaction was up to 8 min, the silver dendrite structure was completely formed (Fig. 2e–f). It can be seen from the three-dimensional morphology of the copper foam that the copper foam has a very large specific surface area, which can provide a large number of adsorption sites for the analyte. The silver dendrite structure provides a large number of hot spots, which significantly increase in Raman signal.

### Optimal reaction time and sensitivity testing of SERS substrates

**Fig. 3:**
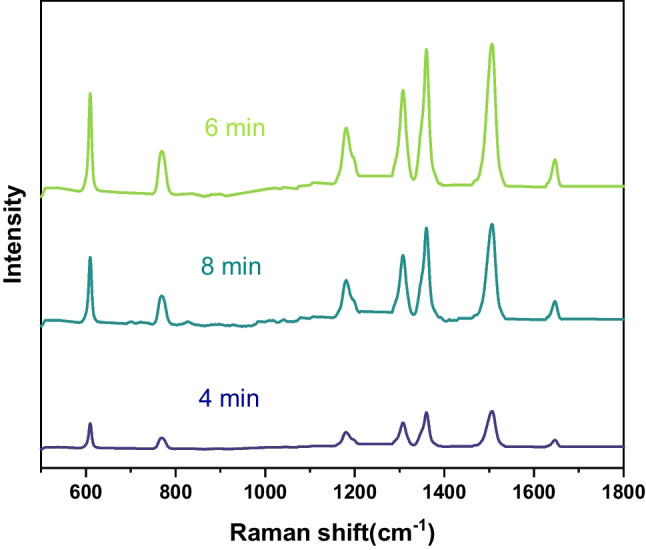
Rhodamine spectrum of SERS substrate with various reaction time

As the reaction time change, the structure of silver nanostructures formed on the surface of copper foam is different. The Ag/Copper foam based SERS substrate with reaction time various from 4 to 8 min was tested for Raman signal sensitivity. R6G was selected as the probe molecule, and the Raman spectra of different reaction times is shown in Fig. 3. It can be seen that the intensity of the Raman characteristic peaks increased from 4 to 6 min, and the intensity of the Raman characteristic peak is decreasing by 8 min.

It is well known that the SERS effect is highly dependent on the number of "hot spots" created by noble metal nanostructures. With the shortening of reaction time, the silver nanostructures are fine and sparse, and the spacing between adjacent nanoparticles is larger. As a result, silver nanostructures cannot provide a large number of hot spots, which leads to the reduction of Raman enhancement. When the reaction time is too long, the silver nanostructures (silver dendrites) become larger and denser, and the surface plasmons of silver branches may not be excited efficiently. Therefore, there is an optimal reaction time for growing silver nanostructures that can provide the greatest number of 'hot spots' to generate the strongest SERS signal. As can be seen from Fig. 3, 6 min’ reaction is the best time for SERS substrate fabrication, so the following tests are all tested with 6 min reaction time SERS substrate.

**Fig. 4:**
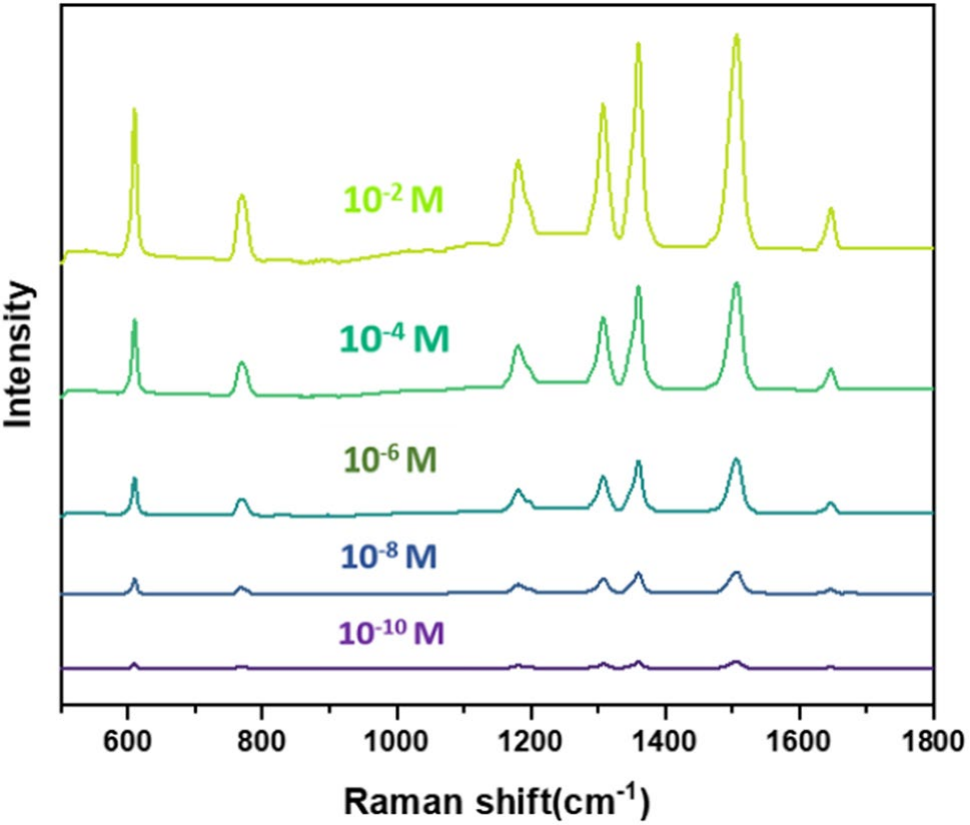
Raman spectrum of 10^–2^–10^−10^ M R6G

Rhodamine was chosen as the probe molecule. The silver nanostructure shows good surface enhancement ability, as shown in Fig. 4. The lower detection limit of R6G is as low as 10^–10^ M, as shown in Fig. 4. The surface-enhanced Raman spectra of R6G at different concentrations were analyzed, and the changes at 610, 770, 1181, 1307, 1359, 1506, and 1644 cm^−1^ were obtained with different concentrations. The peaks at these frequency shifts were all similar to those of Rhodamine molecules. It is related to the stretching vibration of chemical bonds, and the signal intensity increases with the increase of R6G concentration. When the concentration of R6G is 10^–10^ M, the corresponding Raman characteristic peaks can still be found on the graph. The enhancement factor (EF) can be calculated using the Raman intensity of R6G in this experiment, and the calculation formula is:

$${\text{EF}} = \frac{{C_{{{\text{RS}}}} \times I_{{{\text{SERS}}}} }}{{C_{{{\text{SERS}}}} \times I_{{{\text{RS}}}} }},$$

where *I*_SERS_ and *I*_RS_ is the Raman spectral intensity collected in surface-enhanced Raman spectroscopy (Rhodamine 6G + Ag) and Raman spectroscopy (Rhodamine 6G only). In this paper, the enhancement factor is calculated using R6G concentration of 10^–6^ mol/L and Raman shift peak at 1506 cm^−1^. Different enhancement factors reported in other literature should be compared at the same glucose concentration level and the same laser wavelength. Finally, the enhancement factor of the substrate is calculated to be 1.25 × 10^7^. The high EF is attributed to the existence of nano gaps between the silver dendrite nanoparticles on the substrate, which can generate more hot spots.

### Repeatability and stability testing of SERS substrates

**Fig. 5:**
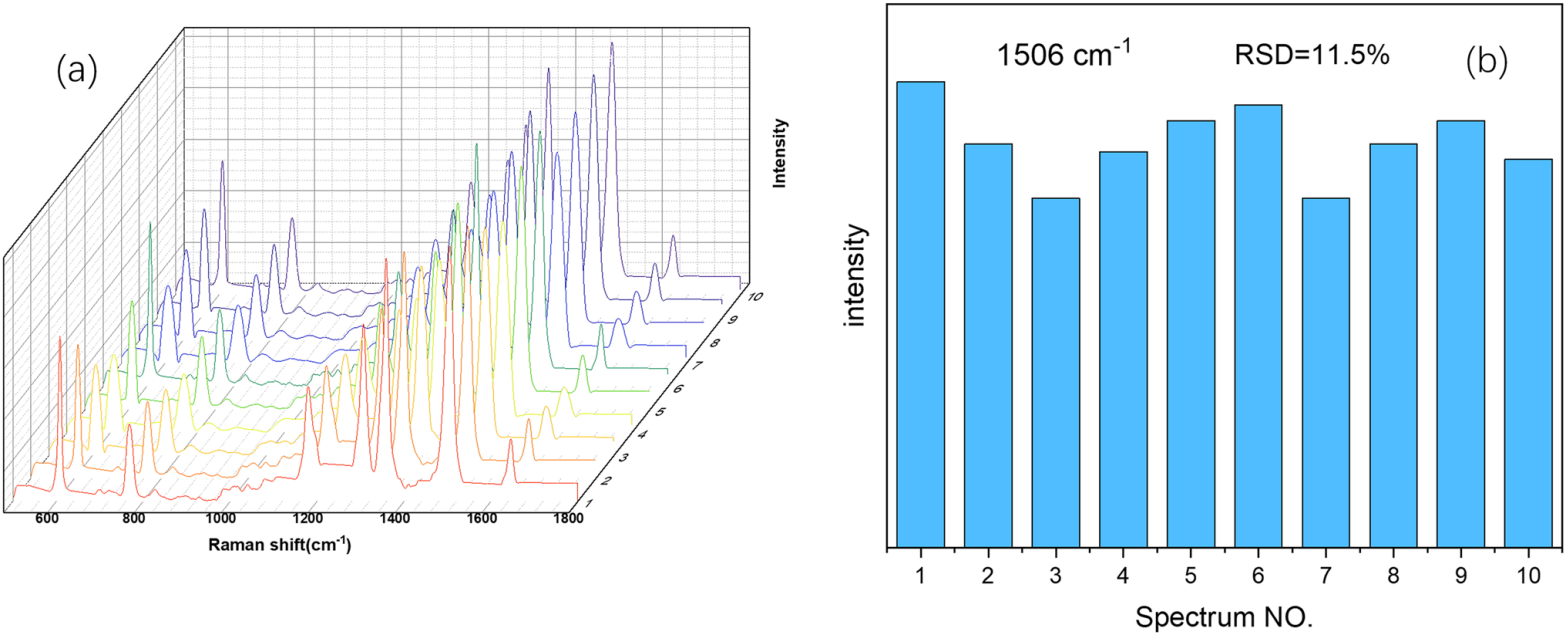
**a** Raman spectrum of 10 points on the substrate and **b** intensity values of 10 points at 1506 cm^−1^

**Fig. 6:**
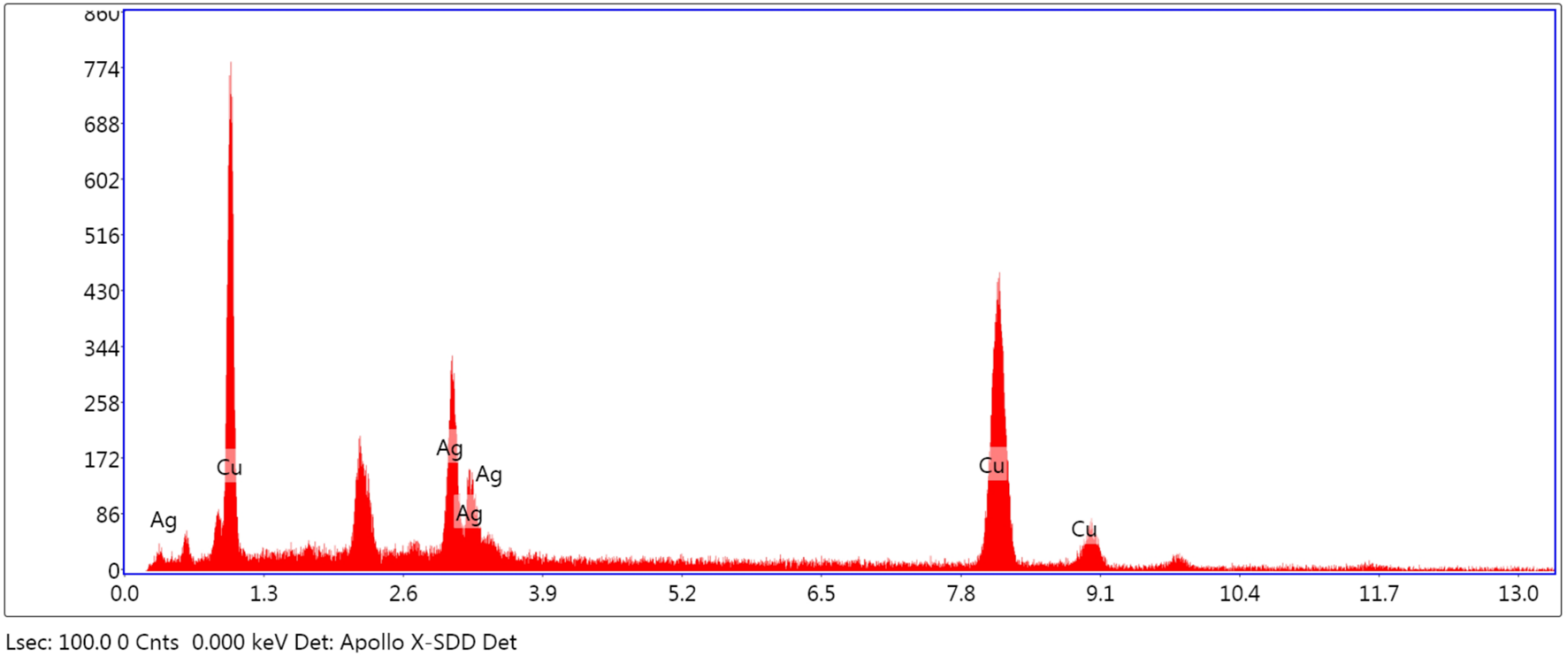
EDS picture of Cu–Ag substrate

Repeatability and stability are also important criteria for evaluating SERS active substrates. The prepared SERS substrates were immersed in 0.1 mM R6G solution for 1 min, so that the R6G molecules were adsorbed on the silver dendrite structure, and the enhanced performance of which could be better utilized. Surface-enhanced Raman spectra of R6G molecules were recorded at 10 random selected spots on the substrate, as shown in Fig. 5a. It shows 10 Raman spectra corresponding to the above 10 spots, and the intensity value at 1506 cm^−1^ was selected to study the change of Raman intensity value, as shown in Fig. 5b. In order to evaluate repeatability of the substrate, the relative mean deviation (RSD) at 1506 cm^−1^ was calculated, and the value was 11.5%, which did not exceed 15%. It can be concluded that the substrate has good repeatability. In order to test the stability of the substrate, the prepared substrate was placed for several days and then tested using EDS technology, and the detection structure is shown in Fig. 6. Most of the types and contents of the substrate shown in the energy spectrum are Cu and Ag, indicating that the substrate has almost no oxidation after several days of placement. It indicates that the substrate has good stability and is suitable for long-term detection.

### Determination of glucose based on SERS

**Fig. 7:**
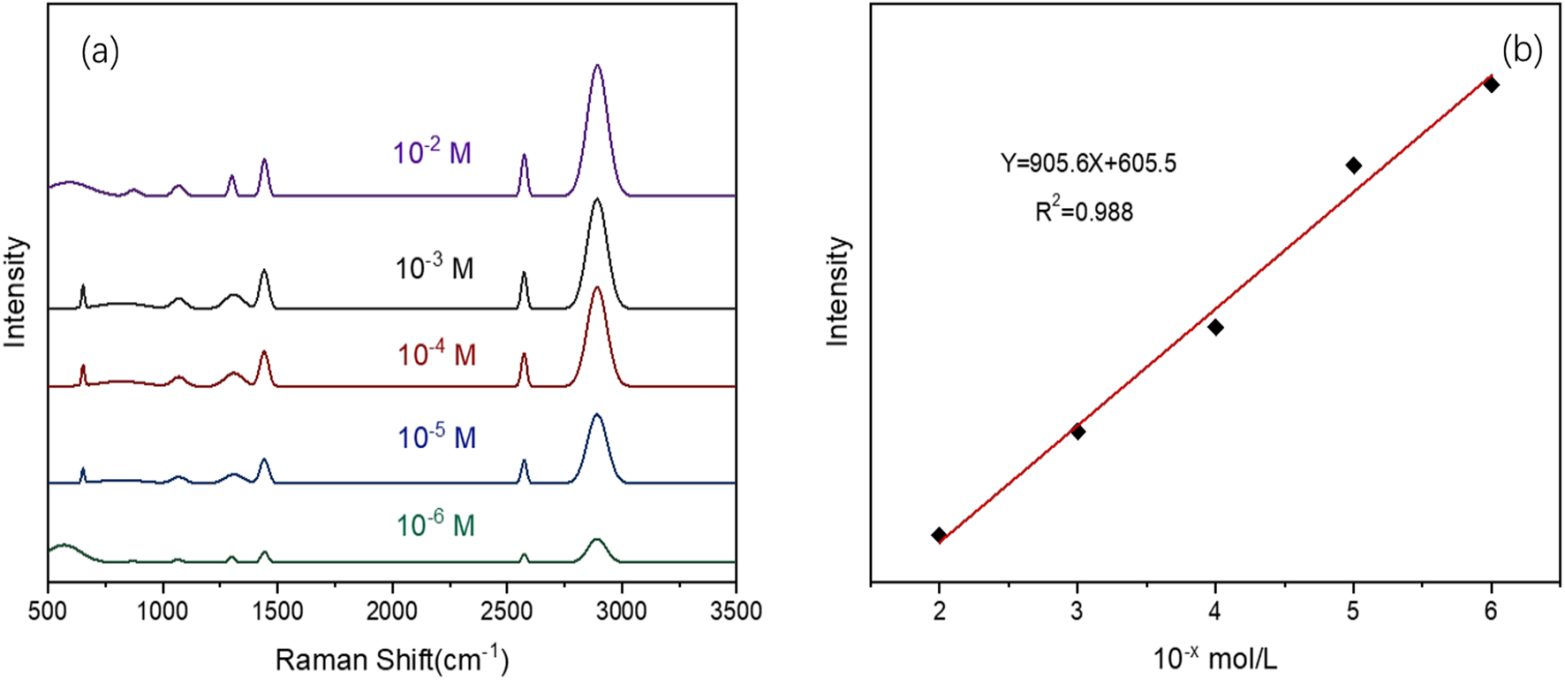
Glucose detection spectrum

Surface-enhanced Raman spectroscopy has shown great application potential in the sensing of various chemical and biomolecules due to its high sensitivity to molecules and abundant structural information. As a physiological disease of human beings, hyperglycemia has received strict attention [39]. Figure 7a shows the surface-enhanced Raman spectra of glucose at different concentrations. The Raman peak intensity increases with the increase of glucose concentration, and the limit of detection is 10^–6^ mol. Table 1 shows the chemical bond stretching vibrations corresponding to different Raman shifts of glucose, which are basically consistent with the published data. Figure 7b shows the relationship between Raman intensity and glucose concentration at the Raman frequency shift of 1440 cm^−1^, with the equation of *Y* = 905.6*X* + 605.5 (*R*2 = 0.988), which proves that there is a good linear relationship between Raman intensity and glucose concentration.

**Table 1:** Documented Raman SERS spectra of glucose and peak assignments

No.	Experimental results/cm^−1^	Peak assignment
1	654	C–C–O bending
2	892	C–C–O in-plane expansion
3	1124	C–C–O out of plane expansion
4	1297	C–H deformation
5	1440	C–H_2_O deformation
6	2584	CH_2_, CH_3_ telescopic vibration
7	2849	CH_2_,CH_3_ telescopic vibration

**Fig. 8:**
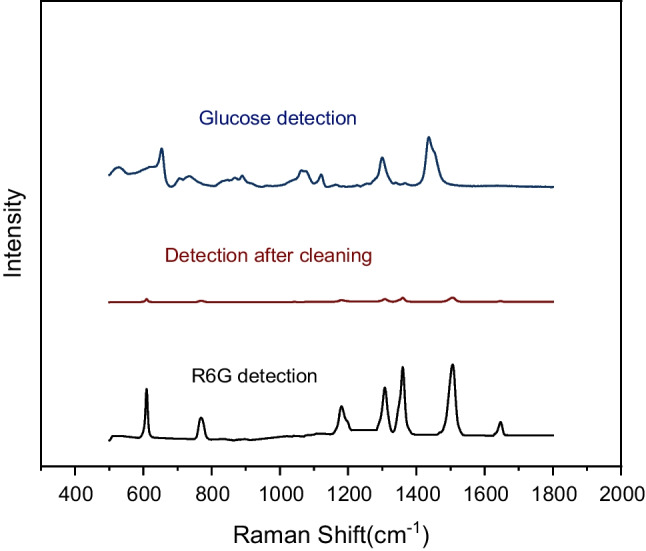
R6G and glucose reutilization test

Reusability is also an important indicator to measure SERS substrate. First, use SERS substrate to detect R6G, then use deionized water to clean the substrate after detection, directly detect the cleaned substrate, and then detect glucose on the substrate. The detection results are shown in Fig. 8. After the detection of R6G, the direct detection of the substrate showed that there was only a small amount of R6G on the substrate. When glucose was detected again, glucose could still be obviously detected from the Raman characteristic peak of glucose, so the substrate had a certain repeatability.

## Conclusions

In conclusion, SERS substrates with good performance were fabricated by the replacement reaction of foamed copper and silver nitrate with a concentration of 10 mmol/L for 6 m. Using R6g as the Raman probe molecule, the limit of detection of this SERS substrate reaches 10^–10^ M. The enhancement factor measured by glucose concentration contrast test was 1.75 × 10^7^. The substrate is very repeatable, with a relative mean deviation of 11.5% at 1506 cm^−1^ as determined by the R6G test. After a few days, the EDS technology was used to detect the material type of the substrate, indicating that the substrate was not oxidized. At the same time, the Raman-enhanced substrate has a limit detection concentration of 10^–6^ M in glucose detection, which has the potential to be suitable for human blood glucose detection. These results show that the SERS substrate prepared based on the foamed copper-silver nitrate replacement reaction has good sensitivity. The SERS substrate is expected to play an important role in the field of biochemical analysis.

## Data Availability

All data generated or analyzed during this study are included in this published.

## Abbreviations

SERS: Surface-enhanced Raman scattering
DT: Mixed decanethiol
MH: Mercaptohexanol
EF: Enhancement factor
